# Interactions Among lncRNA/circRNA, miRNA, and mRNA in Musculoskeletal Degenerative Diseases

**DOI:** 10.3389/fcell.2021.753931

**Published:** 2021-10-11

**Authors:** Yi-Li Zheng, Ge Song, Jia-Bao Guo, Xuan Su, Yu-Meng Chen, Zheng Yang, Pei-Jie Chen, Xue-Qiang Wang

**Affiliations:** ^1^Department of Sport Rehabilitation, Shanghai University of Sport, Shanghai, China; ^2^The Second School of Clinical Medicine, Xuzhou Medical University, Xuzhou, China; ^3^Department of Rehabilitation Medicine, Shanghai Shangti Orthopaedic Hospital, Shanghai, China

**Keywords:** degenerative musculoskeletal disorders, aging, age-related disease, non-coding RNAs, miRNA, circRNA, lncRNA

## Abstract

Musculoskeletal degenerative diseases (MSDDs) are pathological conditions that affect muscle, bone, cartilage, joint and connective tissue, leading to physical and functional impairments in patients, mainly consist of osteoarthritis (OA), intervertebral disc degeneration (IDD), rheumatoid arthritis (RA) and ankylosing spondylitis (AS). Long non-coding RNAs (lncRNAs) and circular RNAs (circRNAs) are novel regulators of gene expression that play an important role in biological regulation, involving in chondrocyte proliferation and apoptosis, extracellular matrix degradation and peripheral blood mononuclear cell inflammation. Research on MSDD pathogenesis, especially on RA and AS, is still in its infancy and major knowledge gaps remain to be filled. The effects of lncRNA/circRNA-miRNA-mRNA axis on MSDD progression help us to fully understand their contribution to the dynamic cellular processes, provide the potential OA, IDD, RA and AS therapeutic strategies. Further studies are needed to explore the mutual regulatory mechanisms between lncRNA/circRNA regulation and effective therapeutic interventions in the pathology of MSDD.

## Introduction

Musculoskeletal degenerative diseases (MSDDs) are pathological conditions that affect muscle, bone, cartilage, joint and connective tissue, leading to physical and functional impairment in patients (Chen Y. et al., 2017; Huo et al., 2018). With the acceleration of the global aging process, the prevalence of MSDD is increasing. This is a huge challenge for patients and healthcare workers, and adds to the global healthcare burden (Li and Chen, 2019). The main MSDD consists of osteoarthritis (OA), intervertebral disc degeneration (IDD), rheumatoid arthritis (RA), and ankylosing spondylitis (AS) (Vinatier et al., 2016; Huo et al., 2018; Loef et al., 2018). OA is a chronic age-related MSDD, featuring for subchondral bone thickening, articular cartilage degradation, and osteophyte formation (Loeser et al., 2012; Hunter and Bierma-Zeinstra, 2019). IDD is also age-related and is caused by progressive degeneration of the disk (Yang S. et al., 2020), causing loss of disk height, reduced hydration and decreased potential to absorb load (Samartzis et al., 2011; Cooper et al., 2016). RA is an autoimmune disease characterized by aggressive arthritis that can lead to joint deformities and loss of function (Smolen et al., 2016). AS, a rare but clear cause of chronic back pain, is an inflammatory disease involving the spine, sacroiliac joints and other joints (Taurog et al., 2016). OA and IDD became mainly responsible for MSDD. Their common character is the broken dynamic equilibrium between catabolism and anabolism in the extracellular matrix (ECM). On the one hand, chondrocytes is only resident cells in the articular system, the ECM degeneration in OA is leaded by chondrocytes’ catabolic and abnormal differentiation (Zhou Z.B. et al., 2019). Cartilage cellularity is reduced in OA because of chondrocyte death. On the other hand, ECM breakdown and abnormal matrix synthesis in IDD is responsible by nucleus pulposus (NP) cells, which are predominant cells in the NP tissue (Fontana et al., 2015). Excessive apoptosis of NP cells could accelerate IDD progression (Zhao et al., 2006). Meanwhile, endplate cartilage degeneration is another risk factor of IDD (Iwakura et al., 2013) due to its irreplaceable nutrition supplement of intervertebral disk (Yuan et al., 2015). Although multiple factors are involved in the pathogenesis of MSDD (Li and Chen, 2019), the development of molecular mechanism of MSDD is still poor. Thus, it is urgent to discover new biomarkers to optimize MSDD early diagnosis and treatment.

With the development of sequencing technology, recent advances have shown that about 98% of the human genome is composed of non-coding RNAs (ncRNAs). In the past, ncRNAs were thought to act as ‘evolutionary junk.’ However, an increasing amount of evidence reported that ncRNAs play an important role in biological regulation (Beermann et al., 2016; Vieira et al., 2018). The main types of ncRNAs include long non-coding RNA (lncRNA), circular RNA (circRNA) and microRNA (miRNA) (Beermann et al., 2016). Recently, extensive evidence suggested that ncRNAs play a vital role in the development of MSDD (Chen W.K. et al., 2017; Yu and Sun, 2018; Wang J. et al., 2019). Moreover, circRNA and lncRNA can interact with miRNA to further regulate downstream target mRNA in the MSDD and play regulatory roles in numerous biological functions, such as proliferation, apoptosis and inflammation. In this review, we focused on the role of lncRNA/circRNA-miRNA-mRNA axis in the development of MSDD and further explored related molecular mechanism of MSDD.

## Interactions Between lncRNA/circRNA and miRNA

### Interactions Between lncRNA and miRNA

MicroRNAs are encoded by endogenous genes, are approximately 20 nucleotides in length and are non-coding single-stranded RNA molecules (Beermann et al., 2016). Since they were first described in *Caenorhabditis elegans*, the number of miRNAs that have been found in mammals increased (Lee et al., 1993). miRNA is evolutionarily conserved and regulates gene expression at the post-transcriptional level by interfering with mRNA translation and degradation (Zhang et al., 2020b). With the iteration of gene chip and sequencing technology, numerous miRNAs have been found to play important roles in MSDD and can be used as biomarkers for clinical diagnosis and treatment (Satoshi Yamashita, 2012; Seeliger et al., 2016; Moran-Moguel et al., 2018). lncRNAs refers to non-protein-coding transcripts with the lengths of more than 200 nucleotides (Beermann et al., 2016). According to the position of the protein-encoding genes in the genome, lncRNAs are divided into five types, namely, intronic, intergenic, bidirectional, sense and antisense (Wang J. et al., 2019). More and more evidence argues that lncRNAs can act as an enhancer or suppressor to regulate the immune response at the epigenetic level, function as scaffold molecules through interactions with RNA-binding proteins in chromatin remodeling complexes (Mathy and Chen, 2017), and then, are involved in many cellular and biological processes in MSDD, such as proliferation, apoptosis, differentiation, inflammation and ECM degradation (Jiang et al., 2017; Li Z. et al., 2018; Abbasifard et al., 2020). Thus, it is important to develop lncRNA as a biomarker and therapeutic target for MSDD.

In recent years, extensive evidence has shown that lncRNAs can interact with miRNAs through several post-transcriptional mechanisms, and the four mechanisms of interaction are as follows. (1) lncRNAs act as miRNA sponges. The lncRNA that can prevent miRNA from acting on mRNA is called competing endogenous RNAs (ceRNAs). These lncRNAs have similar miRNA targets, and they can act as sponges of miRNA, thereby reducing the expression of miRNA and enhancing the translation of target mRNA. lncRNA AK048451 was first considered as an endogenous sponge of miR-489 that can combine with and inhibit the expression of miR-489 (Huang, 2018). (2) Several lncRNAs could directly compete with miRNAs to bind with mRNAs, thereby removing the regulatory roles of miRNAs on mRNAs. For example, lncRNA BACE1AS competes with miR-485-5p to combine with BACE1 mRNA. Thus, the degradation of BACE1 induced by miR-485-5p was inhibited (Faghihi et al., 2010). (3) miRNAs aim at lncRNAs to decrease the stability of lncRNAs and affect the abundance of lncRNAs. It has been verified that lncRNA-p21 was modulated by miRNA let-7b. Upregulation of let-7b promoted the degradation of RNA, leading to the instability of lncRNA-p21 (Deng et al., 2016). (4) Several lncRNAs could generate miRNAs. For instance, lncRNA H19 can generate miR-675 (Deng et al., 2016). To achieve a better understanding of the molecular mechanisms in MSDD progression, in-depth studies about the effects of lncRNAs and their potential downstream miRNA regulators have been performed in recent years.

### Interactions Between circRNA and miRNA

As endogenous RNAs, circRNAs are characterize by covalent loop structures without 5′–3′ polarity nor a polya- denylated tail (Zhou et al., 2018). Different from linear RNA, circRNAs are inherently conserved due to their closed covalent structure and resistance to exonuclides; they are considered to be stable in exosomes (Haque and Harries, 2017). circRNAs are classified into four types according to their origin, namely, exonic circRNAs, exon-intron circRNAs, intronic circRNAs and intergenic circRNAs (Deng et al., 2016). A growing number of studies indicate that circRNAs exist miRNA complementary binding sites to interact with miRNAs, thereby playing regulatory roles in diseases and effecting in many biological processes, such as inflammation, apoptosis and ECM degradation, by participating in the modulation of transcriptional and post-transcriptional levels (Rong et al., 2017; Verduci et al., 2019). The mechanisms included circRNAs acting as miRNAs sponges and miRNAs regulating circRNAs (Kulcheski et al., 2016). For instance, the circAnks1a could regulate VEGFB (vascular endothelial growth factor-B) expression to suppress the excitability of spinal cord by sponging miR-324-3p in neuropathic pain (Zhang S.B. et al., 2019). Pan et al. (2019) elucidated that the miR-1224 could mediate circRNA-Filip1l expression through regulating Ubr5 in the spinal cord of chronic inflammatory pain mice. Although circRNAs are generally considered as ncRNAs because of non-linear structure, several circRNAs, such as CircFBXW7 (Ye et al., 2019) and Circ-EGFR (Liu et al., 2021), are proved to have translation functions due to its translatable open reading frame containing a start codon. The cap-independent translation pathway is thought to be the main mechanism of circRNA translation to encode protein (He et al., 2021). Combined with the above explanation, currently known that circRNAs can interact with proteins or act as miRNA sponges and regulate the expression of upstream gene to participate in the process of diseases development. In recent years, circRNAs have become a research hotspot in MSDD and showed great potential as biomarkers and therapeutic targets (Li H.Z. et al., 2018; Lei B. et al., 2019; Wu et al., 2019).

## Interactions Among lncRNA, miRNA, and mRNA in Degenerative Musculoskeletal Diseases

### Osteoarthritis

In the past decade, quite number of studies have shown that the interaction between lnRNAs and miRNAs is involved in the multiple biological processes of OA, such as inflammation, proliferation, apoptosis, autophagy, cell viability and ECM degradation (Table 1). The major interaction mechanism between lncRNA and miRNA in OA was that lncRNAs as ceRNAs acts as miRNAs sponges. Wang Q. et al. (2017) reported that the expressions of lncRNA OPN and NEAT1 significantly increased, whereas that of miR-181c decreased. According to luciferase assays, miR-181c could combine with NEAT1 and 3′UTR of OPN in synoviocytes, leading to NEAT1 competing with OPN for binding with miR-181c and further enhancing the level of OPN. Chen Y. et al. (2020) showed that lncRNA HOTAIR (HOX transcript antisense intergenic RNA) and mRNA PTEN (phosphatase and tensin homolog) was significantly increased in the OA mice, whereas miR-20b decreased. HOTAIR was involved in the process of apoptosis and ECM degradation by sponging miR-20b and regulating the downstream target PTEN. Lu and Zhou (2020) revealed that lncRNA00662 was downregulated in the cartilage of OA rats. The expression of miR-15b-5p was negative with lncRNA00662, whereas the expression of GPR120 was positively correlated with lncRNA00662. lncRNA00662 regulated GPR120 in apoptosis by serving as a sponge for miR-15b-5p. Sun P. et al. (2020) also studied the effect of XIST on OA patients and showed that XIST upregulated SGTB and inhibited the depression on SGTB induced by miR-142-5p through sponging miR-142-5p. Another study reported that the level of lnc00623 and HRAS was downregulated, whereas miR-101 was increased in OA tissues compared with normal tissues (Lü et al., 2020). Based on luciferase reporter, miR-101 could combine with lnc00623 and HRAS. lnc00623 sponges miR-101 through competing with HRAS, thereby preventing the miR-101-induced depression on HRAS. Some other lncRNAs act as miRNAs sponges in OA and more detailed information is presented in Table 1.

**TABLE 1:** lncRNA/miRNA/mRNA networks in osteoarthritis.

	Species	Diseases	Region	lncRNA	Change	miRNA	Expression	Target gene	Change	Functions	References
(1)	Human	OA	Cartilage	H19	Up	miR-675	Up	COL2A1	Up	Inflammation	Steck et al., 2012
(2)	Human, mice	OA	Cartilage, chondrocyte	GAS5	Up	miR-21	Down	MMPs, ADAMTS-4	Up	Cell apoptosis and autophagy	Song et al., 2014
(3)	Human	OA	Cartilage, chondrocyte	lncRNA-MSR	Up	miRNA-152	Down	TMSB4	Up	ECM degradation	Liu et al., 2016a
(4)	Human	OA	Cartilage, chondrocyte	UFC1	Down	miR-34a	Up	–	–	Cell proliferation and apoptosis	Zhang et al., 2016
(5)	Human	OA	Chondrocyte, C28/I2 cells	HOTAIR	Up	miR-17-3p	Down	ETV1	Up	Cell apoptosis and inflammation	Chen H. et al., 2017
(6)	Human	OA	Cartilage, chondrocyte	lncRNA PVT1	Up	miR-488-3p	Down	–	–	Cell apoptosis	Li Y. et al., 2017
(7)	Human	OA	Cartilage, chondrocyte	lncRNA CIR	Up	miR-27	Down	MMP13	Up	ECM degradation	Li Y.F. et al., 2017
(8)	Human	OA	Cartilage, chondrocyte	lncRNA -UCA1	Up	miR-204-5p	Down	MMP13	Up	Cell proliferation	Wang G. et al., 2017
(9)	Human	OA	Synovium tissues, synoviocytes	NEAT1	Up	miR-181c	Down	OPN	Up	Cell proliferation	Wang Q. et al., 2017
(10)	Rats	OA	Cartilage, chondrocyte	lncRNA MEG3	Down	miR-16	Up	SMAD7	Down	Cell proliferation and apoptosis	Xu and Xu, 2017
(11)	Human	OA	Cartilage, chondrocyte	lncRNA FOXD2-AS1	Up	miR-206	Down	CCND1	Up	Cell proliferation and apoptosis	Cao et al., 2018
(12)	Human	OA	Cartilage, chondrocyte	DANCR	Up	miR-577	Down	SphK2	Up	Cell proliferation and apoptosis	Fan et al., 2018
(13)	Human	OA	Cartilage, chondrocyte	HOTAIR	Up	miR-17-5p	Down	FUT2	Up	Cell proliferation, apoptosis and ECM degradation	Hu et al., 2018
(14)	Human	OA	Cartilage, chondrocyte	XIST	Up	miR-211	Down	CXCR4	Up	Cell proliferation and apoptosis	Mohammadi et al., 2018
(15)	Human	OA	Cartilage, chondrocyte	MALAT1	Up	miR-127-5p	Down	PI3K/Akt	Up	Cell proliferation	Liang et al., 2018
(16)	Mice	OA	Cartilage, chondrocyte	lncRNA-KLF3-AS1	Up	miR-206	Down	GIT1	Up	Cell proliferation and apoptosis	Liu et al., 2018
(17)	Human	OA	Cartilage, chondrocyte	lncRNA CIR	Up	miR-130a	Down	Bim	Up	Cell apoptosis and inflammation	Lu Z. et al., 2019
(18)	Murine	OA	Chondrogenic ATDC5 cells	MALAT1	Up	miR-19b	Down	Wnt/β-catenin and NF-κB pathways	Up	Cell apoptosis and inflammation	Pan et al., 2018
(19)	Human	OA	Cartilage, chondrocyte	lncRNA SNHG5	Down	miR-26a	Up	SOX2	Down	Cell proliferation	Shen et al., 2018
(20)	Human	OA	Human cartilage ATDC5 cells	lncRNA RP11-445H22.4	Up	miR-301a	Down	CXCR4	Up	Cell viability, apoptosis and inflammation	Sun et al., 2018
(21)	Human	OA	Cartilage, chondrocyte	lncRNA -p21	Up	miR-451	Down	–	–	Cell apoptosis	Tang L. et al., 2018
(22)	Human	OA	Cartilage, chondrocyte	lncRNA TUG1	Up	miR-195	Down	MMP13	Up	ECM degradation	Tang L.P. et al., 2018
(23)	Human	OA	ATDC5 cell	MEG3	Down	miR-203	Up	Sirt1	Up	Cell viability, apoptosis and inflammation	Wang et al., 2018e
(24)	Human	OA	Cartilage, chondrocyte	lncRNA DANCR	Up	miR-216a-5p	Down	JAK2/STAT3 signal pathway	Up	Cell proliferation, apoptosis and inflammation	Zhang et al., 2018
(25)	Human	OA	Cartilage, chondrocyte	PVT1	Up	miR-149	Down	–	–	Inflammation	Zhao et al., 2018
(26)	Human	OA	Cartilage, chondrocyte	lncRNA DNM3OS	Down	miR-126	Up	IGF1	Down	Cell proliferation and apoptosis	Ai and Yu, 2019
(27)	Rats	OA	Cartilage, chondrocyte	MEG3	Down	miR-93	Up	TGFBR2	Down	Cell proliferation, apoptosis and ECM degradation	Chen et al., 2019
(28)	Human	OA	Cartilage, ATDC5 cells	lncRNA-HULC	Down	miR-101	Up	NF-κB and p38MAPK signaling pathways	Down	Inflammation	Chu et al., 2019
(29)	Human	OA	Synovial fluid, chondrocytes	MCM3AP-AS1	Up	miR-142-3p	Down	HMGB1	Up	Cell apoptosis	Gao et al., 2019
(30)	Human	OA	LPS-treated C28/I2 cells	H19	Up	miR-130a	Down	–	–	Cell viability, apoptosis, and inflammation	Hu et al., 2019
(31)	Human	OA	Cartilage, chondrocyte	TNFSF10	Up	miR-376-3p	Down	FGFR1	Up	Cell proliferation, apoptosis, and inflammation	Huang et al., 2019
(32)	Human	OA	Chondrocyte	lncRNA SNHG1	Down	miR-16-5p	Up	p38MAPK and NF-κB Signaling Pathways	Down	Inflammation	Lei J. et al., 2019
(33)	Human	OA	LPS-treated ATDC5 cells	MIAT	Up	miR-132	Down	NF-κB and JNK pathways	Up	Cell apoptosis and inflammation	Li et al., 2019a
(34)	Rats	OA	LPS-treated chondrocytes	MALAT1	Down	miR-146a	Up	PI3K	Down	ECM degradation, inflammation and apoptosis	Li et al., 2019b
(35)	Human	OA	Cartilage, synoviocytes	lncRNA-ANRIL	Up	miR-122-5p	Down	DUSP4	Up	Cell proliferation and apoptosis	Li et al., 2019c
(36)	Human	OA	LPS-treated ATDC5 cells	PMS2L2	Down	miR-203	Up	MCL-1	Down	Cell viability, apoptosis, and inflammation	Li et al., 2019d
(37)	Human	OA	Cartilage, chondrocytes	lncRNA-TM1P3	Up	miR-22	Down	ALK1	Up	ECM degradation	Li et al., 2019e
(38)	Human	OA	IL-1β-induced chondrocytes	MALAT1	Up	miR-145	Down	ADAMTS5	Up	ECM degradation	Liu C. et al., 2019
(39)	Murine	OA	LPS-induced ATDC5 cells	THRIL	Up	miR-125b	Down	JAK1/STAT3 and NF-κB pathways	Up	Inflammation	Liu G. et al., 2019
(40)	Human	OA	Cartilages, chondrocytes	PART-1	Down	miR-590-3p	Up	TGFBR2, Smad3	Down	Cell viability and apoptosis	Lu C. et al., 2019
(41)	Human	OA	hMSC, cartilage, chondrocytes	HOTTIP	Up	miR-455-3p	Down	CCL3	Up	Cartilage degradation	Mao et al., 2019
(42)	Human	OA	Chondrocytes	Nespas	Up	miR-291a-3p, miR-196a-5p, miR-23a-3p, miR-24-3p, miR-let-7a-5p	Down	ACSL6	Up	Lipid metabolism	Park et al., 2019
(43)	Human	OA	Synovial fluid, chondrogenic cell line CHON-001	CAIF	Down	miR-1246	Up	IL-6	Up	Cell apoptosis	Qi et al., 2019
(44)	Human	OA	Cartilage, chondrocyte	MEG3	Down	miR-361-5p	Up	FOXO1	Down	Cell proliferation, apoptosis and ECM degradation	Wang A. et al., 2019
(45)	Human, rats	OA	Chondrocyte (Human) cartilage (rat)	XIST	Up	miR-1277-5p	Down	MMP-13, ADAMTS5	Up	ECM degradation	Wang T. et al., 2019
(46)	Human	OA	Cartilage, chondrocyte	FOXD2-AS1	Up	miR-27a-3p	Down	TLR4	Up	Cell proliferation, inflammation and ECM degradation	Wang Y. et al., 2019
(47)	Human	OA	Synovium, chondrocyte	NEAT1	Down	miR-181a	Up	GPD1L	Down	Cell proliferation, apoptosis and inflammation	Wang Z. et al., 2019
(48)	Human	OA	Cartilages, mesenchymal stem cells (MSCs)	HOTAIRM1-1	Down	miR-125b	Up	BMPR2	Down	Cell viability, apoptosis and differentiation	Xiao et al., 2019
(49)	Human	OA	Cartilages, chondrocyte	LINC00341	Down	miR-141	Up	YAF2	Down	Cell apoptosis	Yang Q. et al., 2019
(50)	Murine	OA	LPS-induced ATDC5 cells	lncRNA-ATB	Down	miR-223	Up	MyD88/NF-κB and p38MAPK pathways	Up	Cell viability, apoptosis and inflammation	Ying et al., 2019
(51)	Mice	OA	IL-6-induced ATDC5 cells	CHRF	Up	miR-146a	Down	/	/	Cell viability, apoptosis and inflammation	Yu et al., 2019
(52)	Human	OA	Cartilage, chondrocyte	H19	Up	miR-106a-5p	Down	/	/	Cell proliferation and apoptosis	Zhang X. et al., 2019
(53)	Human	OA	Cartilage, chondrocyte	MALAT1	Up	miR-150-5p	Down	AKT3	Up	Cell proliferation, apoptosis and ECM degradation	Zhang Y. et al., 2019
(54)	Human	OA	Cartilage, chondrocyte	PART1	Up	miR-373-3p	Down	SOX4	Up	Cell proliferation, apoptosis and ECM degradation	Zhu and Jiang, 2019
(55)	Mice	OA	Cartilage, chondrocytes	HOTAIR	Up	miR-20b	Down	PTEN	Up	Cell apoptosis and ECM degradation	Chen Y. et al., 2020
(56)	Human	OA	Cartilage, chondrocytes	HOTAIR	Up	miR-130A-3p	Down	–	–	Cell apoptosis	He and Jiang, 2020
(57)	Human	OA	Cartilage, chondrocyte	GAS5	Up	miR-34a	Down	Bcl-2	Up	Cell apoptosis	Ji Q. et al., 2020
(58)	Rat	OA	BMSCs	BLACAT1	Up	miR-142-5p	Down	–	–	Cell proliferation and differentiation	Ji Y. et al., 2020
(59)	Human	OA	Cartilage, chondrocyte	NEAT1	Up	miR-16-5p	Up	–	–	Cell proliferation and apoptosis	Li D. et al., 2020
(60)	Human	OA	Cartilage, chondrocyte	XIST	Up	miR-376c-5p	Down	OPN	Up	Cell apoptosis	Li L. et al., 2020
(61)	Human	OA	Cartilage, chondrocyte	NEAT1	Up	miR-193a-3p	Down	SOX5	Up	Cell apoptosis, inflammation and ECM degradation	Liu et al., 2020
(62)	Human	OA	Cartilage, chondrocyte	LINC00623	Down	miR-101	Up	HRAS	Down	Cell apoptosis, senescence and ECM degradation	Lü et al., 2020
(63)	Rat	OA	Cartilage, chondrocyte	LINC00662	Down	miR-15b-5p	Up	GPR120	Down	Cell apoptosis	Lu and Zhou, 2020
(64)	Human	OA	Cartilage, LPS-treated C28/I2 cells	MFI2-AS1	Up	miR-130a-3p	Down	TCF4	Up	Cell viability, apoptosis, inflammation and ECM degradation	Luo et al., 2020
(65)	Human	OA	Cartilage, chondrocyte	XIST	Up	miR-142-5p	Down	SGTB	Up	Cell growth, proliferation and apoptosis	Sun P. et al., 2020
(66)	Human	OA	Synovial fluid, chondrocyte	CASC2	Up	miR-93-5p	Down	–	–	Cell apoptosis	Sun Y. et al., 2020
(67)	Human, Rats	OA	Cartilage (human), chondrocyte (rats)	H19	Down	miR-106b-5p	Up	TIMP2	Down	Cell proliferation, migration and ECM degradation	Tan et al., 2020
(68)	Human	OA	Cartilage, chondrocyte	SNHG7	Down	miR-34a-5p	Up	SYVN1	Down	Cell proliferation, apoptosis and autophagy	Tian et al., 2020
(69)	Human	OA	Cartilage, chondrocyte	NKILA	Down	miR-145	Up	SP1	Down	Cell proliferation, apoptosis and inflammation	Xue et al., 2020
(70)	Human	OA	Synovial fluid, chondrocytes	CTBP1-AS2	Up	miR-130A	Down	–	–	Cell proliferation	Zhang et al., 2020a
(71)	Human	OA	Peripheral Blood, THP-1 cell	IGHCγ1	Up	miR-6891-3p	Down	TLR4	Up	Inflammation	Zhang et al., 2020c
(72)	Human	OA	Cartilage, chondrocyte	SNHG15	Down	miR-141-3p	Up	BCL2L13	Down	Cell proliferation, apoptosis and ECM degradation	Zhang et al., 2020e
(73)	Human	OA	Cartilage, chondrocyte	LINC00461	Up	miR-30a-5p	Down	–	–	Cell proliferation, cell cycle progression, inflammation, and ECM degradation	Zhang et al., 2020g
(74)	Human	OA	Cartilage, chondrocyte	OIP5-AS1	Down	miR-29b-3p	Up	PGRN	Down	Cell proliferation, migration, apoptosis and inflammation	Zhi et al., 2020

*ACSL6, acyl-CoA synthetase 6; ADAMTSs, a disintegrin and metalloprotease with thrombospondin motifs; ALK1, activin receptor-like kinase 1; ANRIL, antisense non-coding RNA in the INK4 locus; ATB, activated by transforming growth factor beta; BCL2L13, Bcl2-like 13; Bim, B-cell lymphoma 2 interacting mediators of cell death; BMPR2, bone morphogenetic protein receptor 2; BMSCs, bone marrow stromal stem cells; CASC2, Cancer Susceptibility 2; CCND1, Cyclin D1; CHRF, cardiac hypertrophy-related factor; CIR, cartilage injury–related; CXCR4, C-X-C chemokine receptor-4; DANCR, differentiation antagonizing non-protein coding RNA; DNM3OS, dynamin 3 opposite strand; ECM, extracellular matrix; ETV1, Erythroblast transformation-specific translocation variant 1; FGFR1, fibroblast growth factor receptor 1; FUT2, fucosyltransferase 2; GAS5, Growth Arrest-Specific 5; GIT1, G-protein- coupled receptor kinase interacting protein-1; GPD1L, glycerol-3-phosphate dehydrogenase 1-like; GPR120, G protein−coupled receptor 120; HMGB1, high mobility group protein B1; hMSC, human mesenchymal stem cell; HOTAIRM1-1, HOX antisense intergenic RNA myeloid 1 variant 1; HULC, highly up-regulated in liver cancer; IGF1, insulin-like growth factor-1; JAK1, c-Jun N-terminal kinase 1; LPS, lipopolysaccharide; MALAT1, metastasis associated lung adenocarcinoma transcript 1; MCM3AP-AS1, Minichromosome Maintenance Complex Component 3 Associated Protein Antisense RNA 1; MEG3, maternally expressed gene 3; MEG3, maternally expressed gene 3; MFI2-AS1, melanotransferrin antisense RNA; MIAT, myocardial infarction associated transcript; MMP, matrix metalloproteinase; MSCs, mesenchymal stem cells; MSR, mechanical stress; NEAT1, nuclear enriched abundant transcript 1; NF-κB, nuclear factor κB; OA, osteoarthritis; OIP5-AS1, OIP5 antisense RNA 1; OPN, osteopontin; PART-1, prostate androgen-regulated transcript-1; PGRN, progranulin; PI3K, Phosphoinositide 3-kinase; PMS2L2, PMS1 Homolog 2, Mismatch Repair System Component Pseudogene 2; PVT1, plasmacytoma variant translocation 1; SGTB, small glutamine rich tetratricopeptide repeat containing beta; SNHG, small nucleolar RNA host gene; SOX4, SRY-related high-mobility group box 4; SOX5, Sex-determining region Y-box protein 5; STAT3, signal transducer and activator of transcription 3; TCF4, transcription factor 4; TGFBR2, Transforming growth factor-beta receptor type 2; THRIL, TNF and hnRNPL related immune-regulatory lincRNA; TMSB4, Thymosin β-4; TUG1, taurine upregulated gene 1; UCA1, urothelial carcinoma associated 1; XIST, X-inactive-specific transcript; YAF2, YY1-associated factor 2.*

### Intervertebral Disk Degeneration

**FIGURE 1:**
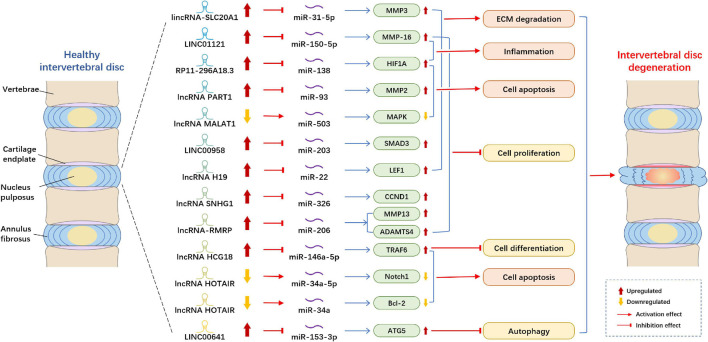
Example of altered lncRNA expression patterns and their biological effects in intervertebral disk degeneration. lncRNA, long non-coding RNA; ECM, extracellular matrix; MMP, matrix metallopeptidase; MAPK, mitogen-activated protein kinase; SMAD3, SMAD family member 3; LEF1, lymphoid enhancing factor-1; CCND1, cyclin D1; ADAMTS4, A disintegrin and metalloproteinase with thrombospondin motifs 4; TRAF6, tumor necrosis factor receptor-associated factor 6; Notch1, Notch Receptor 1; Bcl-2, B cell lymphoma 2; ATG5, autophagy-related gene 5.

The mechanism by which lncRNA and miRNA act on IDD that has been most studied is as follows: lncRNA acts as the sponge of miRNA to modulate target genes (Figure 1). Xi et al. (2017) demonstrated that lncRNA HCG18 was upregulated in the IDD and plays the sponge roles of miR-146a-5p in NP cells. HCG18 is involved in the progression of cell proliferation and apoptosis in NP cells via the miR-146a-5p/TARF6/NF-κB axis. Compared with normal NP tissues, lncRNA SNHG1 (small nucleolar RNA host gene 1) expression was boosted and miR-326, a target gene of SNHG1, was reduced in IDD samples (Tan et al., 2018). Moreover, miR-326 could directly bind with Cyclin D1 (CCND1), and the level of CCND1 in the NP cells markedly increased. Thus, Tan et al. (2018) observed that SNHG1 modulates NP cells proliferation via sponging miR-326 and further regulating CCND1. Another study reported that lncRNA H19 was upregulated in the IDD tissues and could activate Wnt/β-catenin signaling pathway (Wang et al., 2018d). Moreover, miR-326 could directly bind with Cyclin D1 (CCND1), and the level of CCND1 in the NP cells markedly increased. Thus, Tan et al. (2018) observed that SNHG1 modulates NP cells proliferation via sponging miR-326 and further regulating CCND1. Another study reported that lncRNA H19 was upregulated in the IDD tissues and could activate Wnt/β-catenin signaling pathway (Shao et al., 2019). Another research suggested that LINC00641 level increased in NP tissues, whereas miR-153-3p level decreased. ATG5 (autophagy-related gene 5) was a downstream gene of miR-153-3p and upregulated in NP cells (Wang J. et al., 2019). Moreover, LINC00641 could sponge miR-153-3p, and thereby regulate the level of ATG5, cell death and the progression of IDD. Yang Y. et al. (2019) elucidated that lncRNA lincRNA-SLC20A1 (SLC20A1) was overexpressed in IDD patients, and SLC20A1 could induce ECM degradation via sponging miR-31-5p and further modulating the downstream target gene MMP3. Another study established that the level of lncRNA PART1 and mRNA matrix metallopeptidase 2 (MMP2) in NP tissues were significantly higher than those in the control groups, whereas the levels of miR-93 were lower (Gao et al., 2020). Through dual-luciferase reporter assay, they proved that PART1 acts as miR-93 sponges in NP tissues and cells to suppress the expression of miR-93 and to further regulate MMP2. Zheng et al. (2020) showed that MALAT1 was reduced in NP cells, and upregulation of MALAT1 could relieve cell proliferation and apoptosis *in vitro* and inhibit the degree of INN *in vivo*. Moreover, they found that MALAT1 plays pivotal roles in IDD through sponging miR-503, and thereby modulate downstream MAPK signaling pathways.

Several studies indicated that lncRNAs plays roles in IDD by modulating miRNA and their target genes. Wang et al. (2018b) showed that the level of lncRNA-RMRP in degenerated NP tissues was higher than that in normal NP tissues, whereas the expression of miR-206 was lower. They indicated that lncRNA-RMRP could promote cell proliferation via modulating miR-206, thereby regulating downstream target gene MMP13 and ADAMTS4. lncRNA HOTAIR was downregulated in NP tissues and cells, whereas miR-34a expression was negatively correlated with HOTAIR and the expression of Bcl-2 was positively connected with HOTAIR (Yu et al., 2018). HOTAIR could inhibit NP cell apoptosis through regulating miR-34a/Bcl-2 axis. A study found that LINC00958 and mRNA SMAD3 were upregulated in NP tissues, whereas miR-203 was downregulated. Ectopic expression of miR-203 could suppress cell growth and ECM degradation (Zhao et al., 2019). Therefore, LINC00958 participates in the cell process by regulating miR-203 and SMAD3. Another study reported that the expression levels of LINC01121 and MMP-16 significantly increased in NP cells, whereas the level of miR-150-5p decreased (Chen X. et al., 2020). They demonstrated that LINC01121 could enhance the cell process of IDD, such as cell growth, ECM degradation and inflammation by regulating miR-150-5p and MMP-16.

### Rheumatoid Arthritis

In RA disease, the most studied mechanism of lncRNA and miRNA is that lncRNA acts as the miRNA sponge to modulate downstream genes (Table 2). lncRNA PVT1 (plasmacytoma variant translocation 1) and SCUBE2 (signal peptide-CUB-EGF-like containing protein 2) were upregulated, whereas miR-543 was downregulated in synovial tissues of RA rats and patients (Wang et al., 2020). Wang et al. (2020) found that the overexpression of PVT1 or the suppression of miR-543 elevated the level of SCUBE2. Moreover, the knockdown of PVT1 could suppress proliferation and induce apoptosis of RA through hindering the expression of SCUBE2 by sponging miR-543 (Wang et al., 2020). lncRNA LINC-PINT (long intergenic non-protein encoding long-chain RNA p53-induced transcript) was reduced in RA tissues and cells (Wang and Zhao, 2020). Through bioinformatics techniques and RNA Binding Protein Immunoprecipitation (RIP) assay, they found that miR-155-5p could interact with LINC-PINT, and SOCS1 was the target mRNA of miR-155-5p. LINC-PINT could inhibit cell proliferation and invasion via sponging miR-155-5p and regulating the level of SOCS1. Yan et al. (2019) revealed that the level of lncRNA HIX003209 in the peripheral blood mononuclear cells (PBMCs) and macrophages of RA samples and the expression of TLR4 was positively correlated with HIX003209. lncRNA HIX003209 directly targeted miR-6089 and was involved in the regulation of inflammation through acting as miR-6089 sponge via the TLR4/NF-κB signaling pathway.

**TABLE 2:** lncRNA/miRNA/mRNA networks in rheumatoid arthritis and ankylosing spondylitis.

	Species	Diseases	Region	lncRNA	Change	miRNA	Expression	Target gene	Change	Functions	References
(1)	Rat	RA	Synovial tissues	PVT1	Up	miR-543	Down	SCUBE2	Up	Cell proliferation and apoptosis	Wang et al., 2020
(2)	Human	RA	Synovial tissues	LINC-PINT	Down	miR-155-5p	Up	SOCS1	Down	Cell proliferation and invasion	Wang and Zhao, 2020
(3)	Human	RA	Serum	HIX003209	Up	miR-6089	Down	TLR4	Up	Inflammation	Yan et al., 2019
(4)	Human	AS	Serum, fibroblast-like synovial cells	lncRNA MEG3	Down	miR-146a	Up	–	–	Inflammation	Li Y. et al., 2020
(5)	Human	AS	Peripheral blood mononuclear cells	H19	Up	miR675-5p/miR22-5p	miR675-5p up; miR22-5p down	VDR	Up	Inflammation	Zhang et al., 2020f

*AS, ankylosing spondylitis; MEG3, maternally expressed gene 3; PINT, p53-induced transcript; PVT1, plasmacytoma variant translocation 1; RA, rheumatoid arthritis; SCUBE2, signal peptide-CUB-EGF-like containing protein 2; SOCS1, cytokine signaling 1.*

### Ankylosing Spondylitis

That lncRNA acts as the sponge of miRNA to modulate target genes is the most studied mechanism of lncRNA and miRNA acting on AS (Table 2). Li Y. et al. (2020) reported the role of MEG3 (maternally expressed gene 3) in the inflammation of AS. They observed that the expression level of MEG3 in the serum of AS patients was significantly downregulated compared with that in normal people, and MEG3 could inhibit inflammatory responses. However, the expression of miR-146a was upregulated in the AS patients and miR-146a could directly bind with MEG3 (Li Y. et al., 2020). Li Y. et al. (2020) assumed that MEG3 may played a vital role in the repression of inflammation factors in AS through sponging miR-146a, thereby exploring a novel potential treatment target for AS patients. Zhang et al. (2020f) found that lncRNA H19 was highly expressed in the AS patients and elevated the expression level of IL-17A and IL-23 inflammation factors. H19 could directly modulate miR-22-5p and miR-675-5p, and VDR (vitamin D receptor) was the target mRNAs of these two miRNAs. Among them, the level of miR-22-5p was negatively correlated with H19, while miR-675-5p and VDR was positively with H19 in AS patients. H19 plays regulatory roles in inflammatory reaction in AS through binding with VDR by sponging miR-22-5p and interacting with miR-675-5p (Zhang et al., 2020f).

## Interactions Among circRNA, miRNA, and mRNA in Degenerative Musculoskeletal Diseases

### Osteoarthritis

**FIGURE 2:**
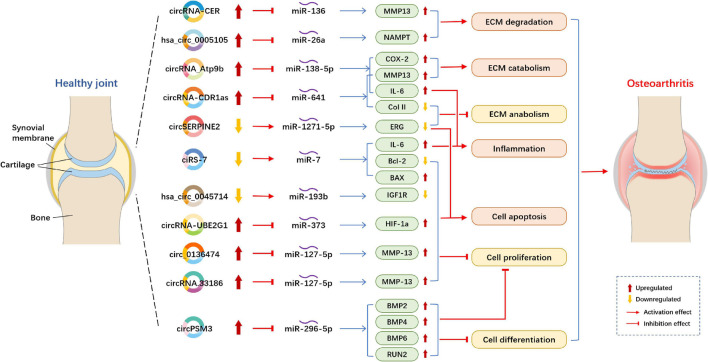
Example of altered circRNA expression patterns and their biological effects in osteoarthritis. circRNA, circular RNA; ECM, extracellular matrix; MMP, matrix metallopeptidase; NAMPT, Nicotinamide phosphoribosyltransferase; COX-2, cyclooxygenase-2; IL-6, interleukin-6; Col II, type II collagen; ERG, E26 transformation-specific-related gene; BAX, BCL2 associated X, apoptosis regulator; Bcl-2, B cell lymphoma 2; IGF1R, insulin-like growth factor 1 receptor; HIF, hypoxia inducible factor; BMP, bone morphogenetic protein.

Circular RNAs acting as miRNA sponges is the one of the most studied mechanisms (Figure 2). Compared with normal cartilage, circRNA-CER (circRNA_100876) was overexpressed and increased with IL-1 (interleukin-1) and TNF-α (tumor necrosis factor-alpha) in OA chondrocytes. circRNA-CER regulated matrix-degrading matrix metalloproteinase (MMP)-13 expression to participated in the process of chondrocyte ECM degradation by sponging miR-136 (Liu et al., 2016b). According to the research of Zhou et al. (2018), overexpressed circRNA_Atp9b sponge miR-138-5p and then mediate ECM catabolism and inflammation to regulates OA progression in chondrocytes by targeting MMP13. circ_0136474 was also verified by the research of Li et al. (2019f) to sponge miR-127-5p to regulate MMP13 in human OA chondrocytes, then, it suppressed cell proliferation and enhanced cell apoptosis during OA progression. The results were in line with those obtained in a study performed by Zhou Z.B. et al. (2019), who found that circRNA.33186/miR-127-5p/MMP13 axis contributes to OA pathogenesis. Furthermore, circSERPINE2 overexpression could slow down the pace of human chondrocytes apoptosis and promote ECM anabolism by sponging miR-1271-5p and thereby targeting ERG (E26 transformation-specific-related gene) to alleviate OA (Shen et al., 2019). In OA blood samples, the downregulation of ciRS-7 and the upregulation of miR-7 were observed (Zhou X. et al., 2019). ciRS-7 was verified to act as a miR-7 sponge to mediate OA progression. Increased cirM3 expression in OA cartilage tissue and cells could serve as a sponge of miR-296-5p to slow down the proliferation and differentiation of OA chondrocytes, thus involving in regulating the occurrence and development of OA chondrocytes (Ni et al., 2020). The overexpression of circRNA-CDR1as regulated OA progression via reducing Col II level but increased IL-6 and MMP13 contents to modulate inflammation and ECM metabolism by sponging miR-641 (Zhang et al., 2020d).

Several circRNA studies showed that circRNAs act as ceRNAs to competitively bind to miRNAs in OA. Hsa_circ_0045714 expression was downregulated (Liu et al., 2016b; Li B.F. et al., 2017). Furthermore, Li B.F. et al. (2017) determined that hsa_circ_0045714 promoted the expression of miR-193b target gene IGF1R (insulin-like growth factor 1 receptor) to regulate chondrocytes proliferation, apoptosis and ECM synthesis. Otherwise, hsa_circ_0005105 expression is significantly enhanced in OA chondrocytes and can promote ECM degradation by mediating the expression of miR-26a target NAMPT (Nicotinamide phosphoribosyltransferase) (Wu et al., 2017). In the lipopolysaccharide (LPS)-induced OA cell model, the expression levels of circRNA-UBE2G1 was significantly increased and bound to miR-373 as ceRNAs to aggravate the OA progression by targeting hypoxia-inducible factor (HIF)-1a (Chen G. et al., 2020).

### Intervertebral Disk Degeneration

**FIGURE 3:**
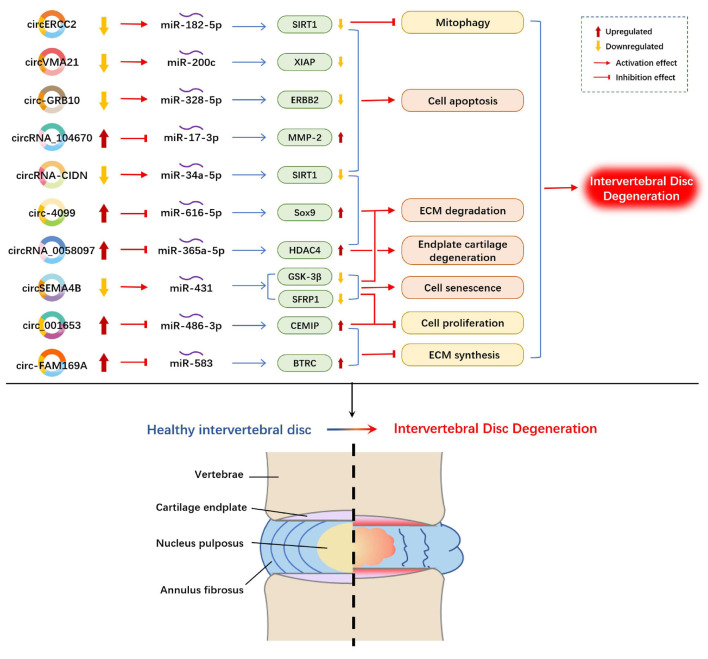
Example of altered circRNA expression patterns and their biological effects in intervertebral disk degeneration. circRNA, circular RNA; ECM, extracellular matrix; SIRT1, silent mating type information regulation 2 homolog 1; XIAP, X linked inhibitor of-apoptosis protein; ERBB2, erb-b2 receptor tyrosine kinase 2; MMP, matrix metallopeptidase; Sox9, SRY-Box transcription factor 9; HDAC4, histone deacetylase 4; GSK-3β, glycogen synthase kinase-3β; SFRP1, secreted frizzled-related protein 1; CEMIP, cell migration-inducing hyaluronan binding protein; BTRC, beta-transducin repeat-containing protein.

Over the past years, some circRNAs have merged as molecular drivers to serve as miRNA sponges or ceRNAs in circRNA/miRNA/mRNA networks in the pathogenesis of IDD (Figure 3). Compared with normal NP tissues, circVMA21 (hsa_circ_0091702) was downregulated in NP tissues and NP cells in IDD and alleviated NP cell apoptosis by targeting miR-200c and XIAP (X linked inhibitor-of-apoptosis protein) (Cheng et al., 2018). Similarly, circ-GRB10 was downregulated during IDD progression, and competitively bound to miR-328-5p to regulate NP cell apoptosis by targeting erb-b2 receptor tyrosine kinase 2 (ERBB2) in the ErbB signaling pathway (Guo et al., 2018). circRNA_104670 was selected via microarray analysis because of its large multiplier expression in IDD tissues (Song et al., 2018). A study reported that circRNA_104670 acted as a ceRNA that binds to miR-17-3p, downregulated circRNA_104670-suppressed MMP-2 expression through circRNA_104670/miR-17-3p/MMP-2 axis, reduced cell apoptosis and increased ECM formation. According to another microarray assay made by Wang et al. (2018a), they selected circ-4099 among 72 upregulated circRNAs in degenerated NP tissues for further analysis. They demonstrated that circ-4099 competitively sponged miR-616-5p, which reversed the suppression of Sox9 by miR-616-5p. Wang et al. (2018c) verified that circSEMA4B was downregulated in IDD specimens, and circSEMA4B served as a miR-431 sponge to compete with SFRP1 or GSK-3β, which are two inhibitory regulators of Wnt signaling, for miR-431 binding, thereby alleviating IL-1β-induced degenerative process in NP cells. circRNA-CIDN was downregulated during IDD progression and bound to miR-34a-5p as a miRNA sponge. Upregulation of miR-34a-5p repressed SIRT1 (silent mating type information regulation 2 homolog 1) to enhance the compression-induced damage of NP cells (Xiang et al., 2020). circERCC2 was also downregulated in IDD NP tissues and NP cells. Furthermore, circERCC2 was associated with the alleviation of IDD through miR-182-5p/SIRT1 axis by activating mitophagy and inhibiting apoptosis (Xie et al., 2019). The expression of circ-FAM169A in IDD samples was significantly upregulated with enhanced ECM catabolism and suppressed ECM anabolism in NP cells. The overexpressed circ-FAM169A competitively bound to miR-583, thus upregulating BTRC (an inducer of the NF-κB signaling pathway) (Guo et al., 2020). circ-FAM169A promoted IDD development via miR-583/BTRC signaling. In addition, circ_001653 could be another novel therapeutic target for IDD that functions by regulating miR-486-3p expression to upregulate CEMIP (cell migration-inducing hyaluronan binding protein) (Cui and Zhang, 2020). circ_001653 downregulation could potentially promote cell proliferation and ECM synthesis through the miR486-3p/CEMIP axis.

Human NP tissues and human endplate tissues were collected to detect differentially expressed circRNAs during IDD progression. Xiao et al. (2020) induced circRNA expression profile changes in endplate chondrocytes, and results reported that 17 circRNAs were upregulated and 12 circRNAs were downregulated (with fold changes higher than 1.5). circRNA_0058097 was selected for further analysis. circRNA_0058097 increased the expression of HDAC4 (histone deacetylase 4) by sponging miR-365a-5p, which intensified the morphological changes of endplate chondrocytes, and aggravated endplate cartilage and ECM degradation.

### Rheumatoid Arthritis and Ankylosing Spondylitis

Rheumatoid arthritis and AS are both characterized by chronic inflammatory disease (van der Heijde et al., 2019; Zhou Y. et al., 2019). However, only a limited number of studies have been conducted on circRNAs in RA and AS (Table 3). Li B. et al. (2018) identified circRNAs in RA synovial tissues and suggested that hsa_circ_0001859 regulated ATF2 expression by competitively sponging miR-204/211. Knockdown of hsa_circ_0001859 suppressed ATF2 expression and decreased inflammatory activity. Hsa_circ_0001859/miR-204/211/ATF2 axis may be used as an approach for treating RA. Another circRNA/miRNA/mRNA network study in RA was conducted by Yang J. et al. (2020), They reported that circRNA_09505 is upregulated in PBMCs from RA patients and mice. The knockdown of circRNA_09505 inhibits macrophage proliferation and alleviates arthritis and inflammation. miR-6089 functions as a ceRNA that is being competitively sponged by circRNA_09505 to regulated macrophage inflammatory response. Furthermore, circRNA_09505 was detected to promote AKT1 expression, which is a direct target of miR-6089, to mediate IκBα/NF-κB signaling pathway. To sum up, circRNA_09505 can sponge miR-6089 and regulate inflammation via miR-6089/AKT1/NF-κB axis in arthritis mice model. Combined with RNA-seq data and RT-qPCR validation of PBMCs from RA patients, the results of Ouyang et al. (2017) showed several upregulated circRNAs (circRNA_101873, circRNA_003524, circRNA_104871, and circRNA_103047), and Wen et al. (2020) proved three upregulated hsa-circRNAs (hsa_circ_0001200, hsa_circ_0001566, and hsa_circ_0003972) and one downregulated hsa_circRNAs (hsa_circ_0008360), but without downstream gene detection to establish circRNA/miRNA/mRNA networks.

**TABLE 3:** circRNA/miRNA/mRNA networks in rheumatoid arthritis.

	Species	Diseases	Region	circRNA	Change	miRNA	Change	Target gene	Change	Functions	References
(1)	Human	RA	Synovial tissues	hsa_circ_0001859	Up	miR-204/211	Down	ATF2	Up	Inflammation	Li B. et al., 2018
(2)	Mice	RA	Peripheral blood mononuclear cells	circRNA_09505	Up	miR-6089	Down	AKT1/NF-κB signaling pathway	Up	Inflammation	Yang J. et al., 2020

*AKT1, threonine kinase 1; ATF2, activating transcription factor 2; NF-κB: nuclear factor κB; RA, rheumatoid arthritis.*

At present, studies on circRNA and miRNA interaction mechanism on AS are lacking. The roles of circRNAs in AS remain unclear. Only one profiling and bioinformatics analysis showed differentially expressed circRNAs in AS patients (sampled form spinal ligament tissues), reported the presence of 57 upregulated circRNAs and 66 downregulated circRNAs in AS spinal ligament tissues (Kou et al., 2020).

Taken together, the study about the interactions among circRNA, miRNA and mRNA in RA and AS may have a great clinical prospect.

## Conclusion and Future Prospect

Recent advances in gene expression of lncRNAs and circRNAs, coupled with the ability to interact with the miRNA, mRNA or signaling pathway, have started to expose the different molecular consequence associated with RNA transcriptions and the roles they play in the development of MSDDs (including OA, IDD, RA, and AS) that involve chondrocyte proliferation and apoptosis, ECM degradation and PBMCs inflammation. The effects of ncRNA/circRNA-miRNA-mRNA axis on MSDD progression elucidated their contribution to the dynamic cellular processes and provided the potential OA, IDD, RA and AS therapeutic strategies. The altered expression of lncRNAs or circRNAs refers to diverse biological processes of MSDD, thereby indicating that lncRNAs/circRNAs may be developed as biomarkers and therapeutic targets. Despite the large numbers of ncRNAs, including lncRNAs and circRNAs, determined to be differentially expressed during these pathogenic processes, only a small portion of them has been elucidated. Research on MSDD pathogenesis, especially on RA and AS, is still in its infancy and major knowledge gaps remain to be filled. Therefore, the interactions among lncRNA/circRNA, miRNA and mRNA in MSDD to present the potential pathogenesis is required. Further studies are needed to explore the mutual regulatory mechanisms between lncRNA/circRNA regulation and effective therapeutic interventions in the pathology of MSDD.

## Author Contributions

X-QW and P-JC: conceptualization and methodology. J-BG, XS, Y-MC, and ZY: investigation. Y-LZ and GS: writing – original draft preparation and writing – review and editing. All authors contributed to the article and approved the submitted version.

## Conflict of Interest

The authors declare that the research was conducted in the absence of any commercial or financial relationships that could be construed as a potential conflict of interest.

## Publisher’s Note

All claims expressed in this article are solely those of the authors and do not necessarily represent those of their affiliated organizations, or those of the publisher, the editors and the reviewers. Any product that may be evaluated in this article, or claim that may be made by its manufacturer, is not guaranteed or endorsed by the publisher.
